# Integrative Multi‐Omics Identifies S100A4 as a Translational Hub Linking Environmental Bis(2‐Ethylhexyl) Phthalate (DEHP) Exposure to Glioblastoma Risk

**DOI:** 10.1155/mi/2385129

**Published:** 2026-02-23

**Authors:** Shasha Tan, Zhou Li, Zhenjiang Du, Jinliang You, Lichun Qiao, Long Zhao, Binbin Yang, Xiaoping Tang, Sajjad Muhammad, Hongjun Liu

**Affiliations:** ^1^ Department of Neurosurgery, The Affiliated Hospital of North Sichuan Medical College, Nanchong, 637000, Sichuan, China, hospital-nsmc.com.cn; ^2^ Department of Neurosurgery, Beijing Anzhen Nanchong Hospital of Capital Medical University and Nanchong Central Hospital, Nanchong, 637000, Sichuan, China; ^3^ Department of Neurosurgery, Medical Faculty, University Hospital Düsseldorf, Heinrich-Heine-Universität, Düsseldorf, 40225, Germany, uni-duesseldorf.de; ^4^ School of Public Health, Xi’an Jiaotong University Health Science Center, Xi’an, 710061, China, xjtu.edu.cn

**Keywords:** DEHP, glioblastoma, lipid metabolic reprogramming, Mendelian randomization, molecular dynamics, multi-omics, S100A4

## Abstract

Glioblastoma (GBM) is a highly aggressive central nervous system malignancy with a dismal 5‐year survival rate of less than 5%, and poorly understood environmental factors complicate its treatment. One such factor is bis(2‐ethylhexyl) phthalate (DEHP, also known as di‐2‐ethylhexyl phthalate), a common plasticizer with documented neurotoxicity, yet its potential role in GBM pathogenesis remains elusive. In this study, we employed an integrative computational framework that combined network toxicology, single‐cell transcriptomics, proteome‐wide and metabolome‐wide Mendelian randomization (MR), and molecular dynamics (MD) simulations to systematically investigate the interplay between DEHP and GBM risk. Cross‐dataset targets were identified by mining toxicogenomic and disease databases, followed by the construction of a protein–protein interaction (PPI) network. This approach identified 76 overlapping targets between DEHP and GBM, which were refined to 24 hub genes through topological analysis. Notably, MR analyses revealed putative causal associations between higher genetically predicted plasma levels of three hub proteins—CD63, CTSS, and S100A4—and an increased risk of GBM, with S100A4 showing the strongest effect (odds ratio [OR] = 2.03, 95% confidence interval [CI] 1.15–3.58, *p* = 0.0149). This association was consistently validated across 11 independent cohorts, including TCGA, GTEx, and GEO datasets. Molecular docking and dynamics simulations identified S100A4 as the predominant binding target of DEHP, revealing a high‐affinity interaction that may stabilize a metastasis‐associated conformation. A two‐step MR mediation analysis further indicated that S100A4 partially influences GBM risk by altering plasma lipid metabolites, with erucic acid mediating ~17% of the total effect. In conclusion, our analysis provides converging computational and genetic epidemiological evidence for a novel DEHP–S100A4–lipid metabolic axis that may contribute to GBM development. This pathway conceptually bridges environmental toxicology and neuro‐oncology and highlights S100A4 and associated lipid disturbances as potential targets for preventive intervention. However, the proposed mechanistic links remain inferential, and definitive confirmation will require future in vitro and in vivo experiments to directly test the impact of DEHP on S100A4 expression, function, and downstream metabolic reprogramming in GBM models.

## 1. Introduction

Glioblastoma (GBM), the most aggressive primary malignant tumor of the central nervous system, remains a devastating disease with limited therapeutic progress over the past two decades. A recent integrative analysis underscores the ongoing challenge and the potential of novel molecular targets, including noncoding RNAs and hub genes, in advancing GBM research [1]. Despite the adoption of a standardized therapeutic regimen that includes maximal safe resection, temozolomide (TMZ) chemotherapy, and radiotherapy, the prognosis for GBM remains dismal, with a median survival of 15 months and a 5‐year survival rate below 5% [2–5]. The clinical efficacy is further limited by several interconnected barriers: the blood–brain barrier (BBB), which restricts ~98% of small‐molecule drugs and nearly all large‐molecule agents, preventing effective drug delivery [6]; profound tumor heterogeneity, which promotes diverse and resilient cell populations; and rapid acquired resistance to TMZ chemotherapy, which enables tumor cells to evade treatment [7, 8]. Moreover, GBM displays profound intratumoral heterogeneity across genomic, transcriptomic, and spatial axes—with coexisting subclones (e.g., EGFRvIII‐positive, TP53‐mutant, and IDH1‐wildtype populations). Single‐cell and spatial multi‐omics studies in recent years have repeatedly demonstrated this heterogeneity and implicated it in therapeutic failure and clonal selection under treatment pressure [9–11]. Resistance to TMZ is a major clinical challenge in GBM. Evidence from clinical cohorts and preclinical models indicates that MGMT expression and promoter methylation status modulate tumor sensitivity to TMZ, contributing to heterogeneous treatment outcomes [12, 13].

Multiple emerging therapeutic modalities—including immune checkpoint inhibitors, molecular targeted agents, nanomedicine, and localized hyperthermia—have demonstrated preclinical promise but largely failed to deliver durable survival benefit in GBM clinical trials. Large randomized trials of immune checkpoint blockade (e.g., CheckMate‐143) did not improve overall survival compared with standard comparators, and numerous targeted‐therapy and nanomedicine approaches have similarly encountered translational hurdles related to tumor heterogeneity, the BBB, and the immunosuppressive tumor microenvironment [14–17].

Bis(2‐ethylhexyl) phthalate (DEHP), a widely used endocrine‐disrupting plasticizer, has been identified as a high‐production‐volume chemical used predominantly in polyvinyl chloride (PVC) plastics and related consumer products [18]. It is ubiquitous in the environment and in human exposure pathways—including dietary (via food packaging materials), inhalational (via indoor air and dust), and dermal contact [19]. DEHP can migrate into lipid‐rich foods, and migration is enhanced by heating and prolonged contact. Indoor dust and air may contain elevated concentrations of DEHP relative to outdoor ambient levels, reflecting significant emissions from indoor plastic and consumer product sources [20]. Vulnerable populations—including neonates treated with PVC‐based medical tubing and children using packaged food and toys—have been shown in biomonitoring studies to carry higher internal burdens of phthalate metabolites than adults [18]. Although occupational cohort studies in PVC‐related industries have reported increased central nervous system tumor mortality, direct epidemiologic evidence linking DEHP exposure specifically to GBM remains sparse. This gap underscores the need to interrogate molecular and mechanistic pathways linking environmental plasticizer exposure to GBM pathogenesis.

Existing toxicological studies are limited by the use of single‐omics profiling, lacking a systems‐level view of DEHP‐driven molecular alterations across GBM‐related signaling pathways, tumor microenvironment remodeling, and metabolic reprogramming. Furthermore, these models rarely incorporate genetic determinants of interindividual susceptibility, which are essential for establishing causal inference and translational relevance.

To address these gaps, we established an integrative multi‐omics framework combining network toxicology, single‐cell transcriptomics (single‐cell RNA sequencing [scRNA‐seq]), proteome‐wide and metabolome‐wide Mendelian randomization (MR), and molecular dynamics (MD) simulations to systematically dissect the mechanistic relationship between DEHP exposure and GBM risk. We first identified 76 overlapping DEHP‐ and GBM‐associated targets across toxicogenomic, disease, transcriptomic, and proteomic datasets, followed by protein–protein interaction (PPI) analysis, which revealed 24 core hub genes. MR analysis using UK Biobank proteomic quantitative trait loci as exposures and FinnGen GBM GWAS as outcomes demonstrated significant causal associations between increased expression of three proteins—S100A4, CD63, and CTSS—and elevated GBM risk, with S100A4 showing the strongest effect (odds ratio [OR] = 2.03, *p* = 0.0149). This causal link was replicated across 11 independent cohorts, including TCGA, GTEx, GEO, and scRNA‐seq datasets, underscoring its robustness. MD simulations further revealed nanomolar‐affinity binding of DEHP to S100A4 (Δ*G* = –37.22 kcal/mol), stabilizing its pro‐metastatic conformation.

Notably, our mediation analysis uncovered a previously unrecognized DEHP–S100A4–lipid metabolic axis. In our conceptual model, S100A4 upregulation may reprogram lipid metabolism in the GBM tumor microenvironment, with erucic acid (22:1n9) accounting for ~17% of the S100A4‐associated increase in GBM risk. This mechanistic axis provides a biologically plausible link between environmental toxicant exposure, high‐affinity binding to oncogenic drivers, and metabolic reprogramming in GBM.

Altogether, this integrative analysis provides mechanistic evidence linking DEHP exposure to GBM development, primarily through computational and genetic epidemiological insights. These results suggest that DEHP may act as a modifiable environmental risk factor, that S100A4 could serve as a therapeutic target to counter exposure‐driven tumorigenesis and lipid metabolism represents a promising axis for intervention. However, these implications are speculative and require rigorous experimental validation. This work informs future precision oncology approaches and public health strategies to reduce environmental contributions to GBM risk.

## 2. Materials and Methods

### 2.1. Study Design

**Figure 1:**
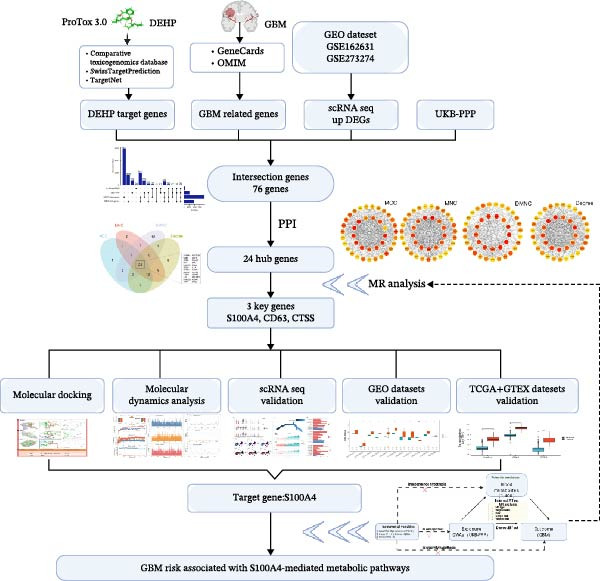
Integrative multi‐omics framework deciphering the DEHP–S100A4–lipid axis in glioblastoma pathogenesis.

This study employed an integrative computational biology framework with a causal inference component to investigate the molecular mechanisms by which the environmental plasticizer DEHP contributes to GBM development (Figure 1). We first performed cross‐analyses of toxicogenomic databases (including ChEMBL [21], SwissTargetPrediction [22], and the Comparative Toxicogenomics Database [23]) and disease databases (including GeneCards [24] and OMIM [25]). These results were then integrated with GBM single‐cell transcriptome data (GSE162631 [26], GSE273274 [27]) and protein quantitative trait loci (pQTL) data from the UK Biobank Pharma Proteomics Project (UKB‐PPP). This integrative process identified 76 cross‐dataset potential targets relevant to both DEHP and GBM. Following a topology analysis of the PPI network, the list was refined to 24 hub genes for subsequent causal inference.

To assess causality, we performed MR analysis using exposure data from the UKB‐PPP and outcome data from the FinnGen cohort (comprising 406 GBM cases and 378,749 controls). Sensitivity analyses, including MR‐Egger regression and Cochran’s Q test, supported the robustness of the findings. This analysis identified S100A4 as the most promising causal protein, with an OR of 2.03 (*p* < 0.05). To explore the physical basis of this association, we conducted molecular docking (AutoDock Vina v1.2.7) and 100‐nanosecond MD simulations (AMBER22/MM‐GBSA). These investigations demonstrated that DEHP stably interacts with the S100A4 protein, exhibiting a high binding affinity (Δ*G* = −37.22 kcal/mol), suggesting that it may stabilize a specific protein conformation.

Furthermore, a two‐step MR mediation analysis was employed to elucidate the downstream metabolic consequences. This revealed that the causal effect of S100A4 on GBM risk is partly mediated through perturbations in plasma lipid metabolites, with erucic acid (22:1n9) accounting for ~17% of the total effect. The transcriptional relevance of this pathway was validated across multiple independent cohorts, including TCGA, GTEx, and 11 GEO datasets. Overall, this study constructs a comprehensive evidence chain linking “environmental exposure → structural interaction → genetic causality → metabolic phenotype,” thereby proposing a novel analytical framework for research into environment‐related tumors. The design and reporting of MR analyses followed the STROBE‐MR guideline [28, 29]. The completed STROBE‐MR checklist is provided as Supporting Information 1: STROBE‐MR checklist.

### 2.2. Toxicological Analysis of DEHP and Collection of Targets

Initially, we retrieved the Simplified Molecular Input Line Entry System (SMILES) string for the compound DEHP from the PubChem database [30]. This sequence was then entered into the ProTox3 database [31] to generate predictive results. At the beginning of our study, we verified the standard nomenclature and molecular formula of DEHP by utilizing the PubChem database. Subsequently, we uploaded molecular files in SDF format to the SwissTargetPrediction platform. We screened all predicted targets with a probability greater than zero, then downloaded the results. These identified targets were preliminarily regarded as potential candidates for interaction with DEHP. At the same time, we gathered potential targets specific to the species “*Homo sapiens*” (humans) from the ChEMBL database. We then integrated the results from CTD and SwissTargetPrediction with the targets obtained from ChEMBL, removing duplicates through a systematic deduplication process. To ensure consistent nomenclature, we standardized the target names using the UniProt database [32]. Ultimately, we completed the merging and deduplication process, resulting in a comprehensive target library for DEHP. All data were retrieved on July 5, 2025.

### 2.3. Construction of Disease Targets

The construction of the disease target library was primarily based on the GeneCards and OMIM databases, with “GBM” as the primary search keyword. To ensure that the genes identified demonstrated a strong association with GBM and to assess the implications of DEHP, only those genes with a GeneCards relevance score exceeding 2 were included in the selection process. Additionally, OMIM targets were incorporated into the library. After merging the data from both sources, any duplicates were eliminated. Ultimately, this process yielded 5122 unique targets, thereby establishing the disease target library. All data were retrieved on July 5, 2025.

### 2.4. Single‐Cell Analysis and Identification of Differentially Expressed Genes

To identify specifically upregulated genes in the macrophage subset within the GBM tumor microenvironment, we performed an integrated analysis of scRNA‐seq data from 14 samples (7 GBM tumors and 7 peritumoral normal tissues) sourced from the GEO database under accessions GSE162631 and GSE273274. We focused on macrophages due to their established role as a dominant immune cell population in GBM, critically shaping the immunosuppressive tumor microenvironment and promoting tumor progression. Identifying DEHP‐responsive genes within this key stromal compartment could reveal mechanisms by which environmental factors modulate the GBM niche. Raw data underwent stringent quality control, excluding genes detected in fewer than three cells, cells with fewer than 250 detected genes, and cells with a mitochondrial gene percentage exceeding 10%. Potential doublets were removed using DoubletFinder (pN = 0.25, pK = 0.09, nExp calculated based on 8% expected doublet rate) before downstream analysis. Data normalization was then carried out using the LogNormalize method. To mitigate batch effects across samples, we applied the Harmony algorithm for integration and correction. Subsequently, principal component analysis (PCA) was performed on the corrected data, followed by cell clustering using the first 30 principal components (resolution = 0.5) and UMAP for visualization. Cell clusters were annotated to major types, including macrophages, based on marker genes identified using the FindAllMarkers function in Seurat (|log_2_FC| ≥0.25, min_pct ≥0.25, adjusted *p*‐value <0.05). Finally, within the annotated macrophage subset, we used the FindMarkers function to directly compare GBM and normal samples, identifying significantly upregulated differentially expressed genes (upDEGs) with the thresholds: log_2_FC >0.5, min_pct >0.25, and adjusted *p*‐value ≤0.05.

### 2.5. Data Sources

We utilized pQTL data from the UKB‐PPP [33], a resource generated from the plasma proteomic profiling of 54,219 UK Biobank participants. This dataset comprises pQTL mappings for 2923 plasma proteins, summarizing 14,287 independent genetic associations, including both cis‐ and trans‐pQTLs, providing a comprehensive genetic perspective on plasma protein abundance.

The GWAS data were obtained from the Finnish FinnGen database, specifically version R12. This initiative, a collaboration between public and private sectors, focused on exploring genotype‐phenotype relationships within the Finnish ancestral population. The analysis included data from 406 patients diagnosed with GBM and 378,749 control samples (dataset ID: finngen_R12_C3_GBM_EXALLC.gz). Furthermore, in 2023, a study listed in the GWAS Catalog contributed additional GWAS data on 1400 plasma metabolites, with identifiers ranging from GCST90199621 to GCST90201020 [34]. This study specifically focused on participants of European descent to reduce population stratification and enhance the validity of the findings.

### 2.6. Construction of the PPI Network

Genes located at the intersection of four datasets—DEHP‐associated targets, GBM disease‐related genes, upDEGs in GBM macrophages, and plasma proteins quantified in the UKB‐PPP—were first identified using the “VennDiagram” package in R. Only genes present in all four sets, and thus simultaneously DEHP‐related, GBM‐related, transcriptionally upregulated in the GBM microenvironment, and instrumented by pQTLs in UKB‐PPP, were retained. This procedure yielded 76 overlapping genes for network analysis.

These 76 genes were submitted to the STRING database [35] (organism: *Homo sapiens*, minimum required interaction score: 0.4) to construct a PPI network, which was then imported into Cytoscape [36] for visualization and topological analysis. Hub genes were prioritized using the CytoHubba plugin, applying four centrality algorithms—Degree, Density of Maximum Neighborhood Component (DMNC), Maximum Neighborhood Component (MNC), and Maximal Clique Centrality (MCC)—with default parameters. For each algorithm, the top 35 ranked genes were extracted, and the intersection of these four top‐35 lists was defined as the final set of robust hub genes for subsequent analyses.

### 2.7. MR Analysis

We conducted a two‐sample MR analysis to infer putative causal effects of circulating plasma protein levels on GBM susceptibility. Genetic instruments for protein exposures were derived from the UKB‐PPP. Analyses were performed using the “TwoSampleMR” package in R, employing the Wald ratio estimator for proteins with a single instrumental variable and the IVW method for those with multiple independent instruments. Effect estimates are presented as OR per one‐standard‐deviation increment in genetically predicted plasma protein levels. All MR effect estimates are reported as OR per one‐standard‐deviation increase in genetically predicted exposure, together with their 95% confidence intervals (CIs) and corresponding *p*‐values.

### 2.8. Molecular Docking

Molecular docking was employed to analyze the intermolecular interactions between DEHP and the core target proteins identified in this study. The three‐dimensional structure of the small‐molecule ligand (DEHP) was obtained from PubChem. Water molecules and the original ligand(s) that were bound to the target proteins were removed using PyMOL. After preparing the protein structures—adding hydrogens, calculating charges, and merging nonpolar hydrogens—the proteins were imported into AutoDock Vina. The grid box dimensions and genetic algorithm parameters were defined based on the active site characteristics, and molecular docking was performed with AutoDock Vina. Visualization of the docking results was performed using Discovery Studio (version: Discovery Studio 2025 Client) and PyMOL 2.5.

### 2.9. MD Simulation

MD simulations were conducted using the AMBER22 software package [37]. The initial configurations utilized were the docked complexes formed between the small molecule and the protein. Before initiating the simulations, the partial charges of the small molecule were determined using the antechamber module and Gaussian09 software with the Hartree‐Fock (HF) SCF/6‐31G theory. The small molecule and protein were subsequently parameterized using the GAFF2 and ff14SB force fields, respectively. Hydrogen atoms were added via the LEaP module, and the system was solvated in a truncated octahedral TIP3P water box (extending 10 Å in each direction). Na^+^/Cl^−^ ions were added to neutralize the system’s charge, which generated the final topology and parameter files.

The MD simulations were performed using the AMBER22 software package. Energy minimization was first performed using 2500 steepest descent steps, followed by 2500 conjugate gradient steps. The system was then heated over 200 ps from 0 to 298.15 K under the NVT ensemble and subsequently equilibrated for 500 ps under NVT to achieve uniform solvent distribution. This was followed by an additional 500 ps equilibration under the NPT ensemble. Finally, a 100 ns production simulation was carried out in the NPT ensemble with periodic boundary conditions to obtain stable trajectory data. The cutoff distance for nonbonded interactions was set to 10 Å, and long‐range electrostatic interactions were calculated using the Particle Mesh Ewald (PME) technique [38]. Hydrogen bonds were constrained using the SHAKE algorithm [39]. Temperature was controlled at 298.15 K using a Langevin thermostat [40] with a collision frequency (*γ*) of 2 ps^−1^; pressure was maintained at 1 atm. A 2 fs integration time step was used, and trajectory snapshots were saved every 10 ps.

### 2.10. Molecular Mechanics‐Generalized Born (GB) Surface Area (MM‐GBSA)

MM‐GBSA is one of the computationally fastest methods for calculating binding free energies using force‐field methods. It achieves this by comparing the free energies of the protein, the ligand, and the complex in solution [41]. The binding free energy of the compound‐target complex was calculated using the GB implicit solvent model input data within AMBER22.

### 2.11. Mediation Analysis and Sensitivity Analysis

To confirm the robustness of our findings, we performed multiple sensitivity analyses. Cochran’s Q test indicated no significant heterogeneity among the genetic variants (*p* > 0.05) [42], and the intercept test from MR‐Egger regression, along with an outlier analysis for pleiotropy, provided no evidence of horizontal pleiotropy (both *p* > 0.05) [43, 44]. Additionally, we conducted a leave‐one‐out study by sequentially excluding each instrumental variable: the MR analysis was repeated after removing each in turn to assess its impact on the overall effect estimate.

Based on these robust results, we further employed an integrated framework combining two‐step MR with mediation analysis. This approach aims to systematically elucidate the association pathways between the target gene and GBM risk, while evaluating the potential mediating role of plasma metabolites in this process. For the plasma metabolites, we utilized an effects decomposition model, breaking down the total effect (*β*_all_) into a direct effect (*β*_direct_), which refers to the effect that is independent of the intermediate variable, and an indirect effect (*β*_1_^∗^ *β*_2_), which refers to the effect mediated by the plasma metabolite indicator, defined as the specific metabolite under investigation. In this study, the proportion of the indirect effect to the total effect (*β*_1_^∗^ *β*_2_/*β*_all_) greater than 10% was set as the threshold for determining whether the mediation effect was statistically significant.

## 3. Results

### 3.1. Single‐Cell Transcriptomic Atlas Reveals Features of the GBM Microenvironment and Key Macrophage‐Associated Genes

Integrated analysis of 14 samples from two independent datasets yielded 147,444 high‐quality cells after stringent quality control. Following doublet removal and Harmony‐based batch‐effect correction, unsupervised clustering of the top 30 principal components at a resolution of 0.5 identified 27 distinct cell clusters. Systematic annotation based on classical marker genes defined 12 major cell types (Figure 2A), with representative marker expression patterns shown in the annotation plot (Figure 2B). Cellular composition analysis revealed macrophages as the dominant population (84,775 cells, 57.5%), with detailed proportional distribution of all cell types presented in the cellular proportion plot (Figure 2C).

Figure 2Single‐cell transcriptomic profiling of the glioblastoma microenvironment and identification of key macrophage‐associated genes. (A) UMAP visualization shows 12 distinct annotated cell types and their proportional distributions. (B) Cell type annotation based on classical marker gene expression. (C) Cellular composition analysis reveals the proportional distribution of major cell types in the GBM microenvironment. (D) Volcano plot displaying differentially expressed genes across major cell types. (E) Scatter plot comparing average expression (log1p‐transformed counts) of representative genes in macrophages from GBM versus peritumoral control tissues. Genes above the diagonal line (*y* = *x*) show higher expression in GBM. Highlighted genes *CD63*, *CTSS*, and *S100A4* represent significantly upregulated transcripts in tumor‐associated macrophages.

**Figure:**
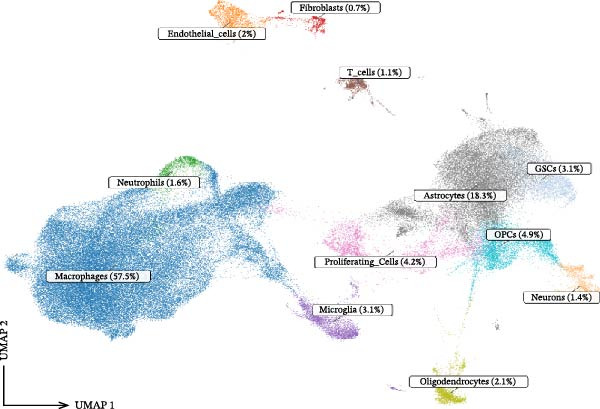
(A)

**Figure:**
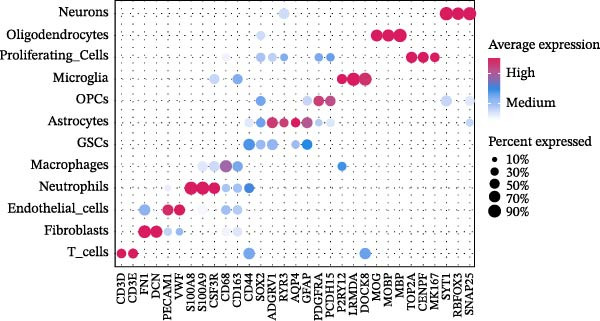
(B)

**Figure:**
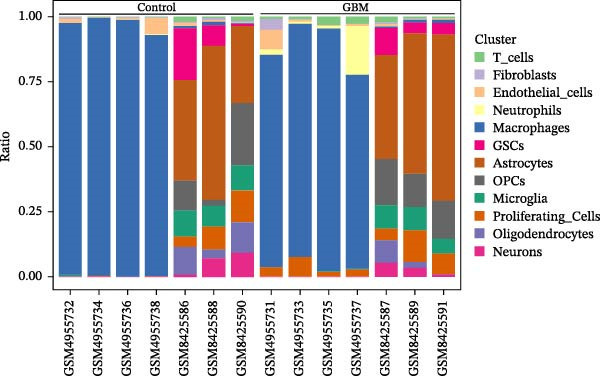
(C)

**Figure:**
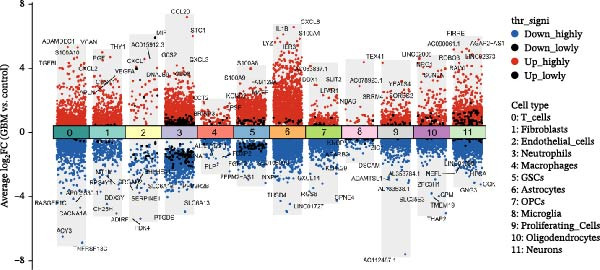
(D)

**Figure:**
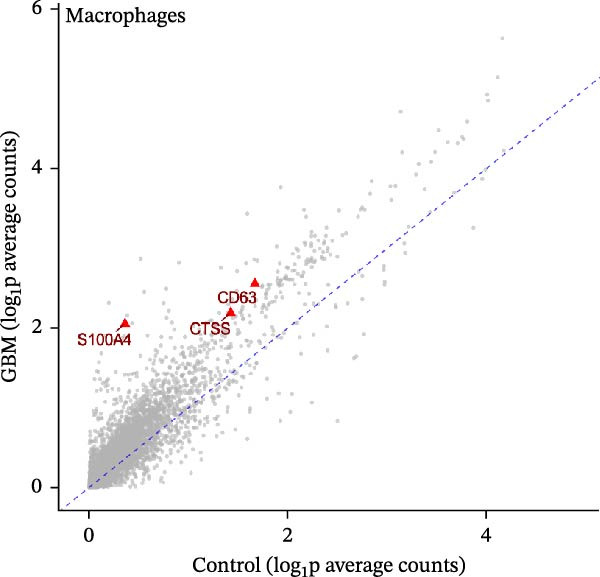
(E)

To elucidate cell‐type‐specific transcriptional alterations in GBM pathogenesis, we performed differential expression analysis across all annotated populations. Volcano plots revealed a substantial number of differentially expressed genes across all 12 annotated cell types (including macrophages, astrocytes, oligodendrocyte precursor cells, etc.) (Figure 2D). Notably, within the macrophage subpopulation, we identified a set of significantly upregulated genes in GBM tissues under stringent criteria (log_2_FC >0.5, min_pct >0.25, adjusted *p*‐value ≤0.05). These macrophage‐specific differentially expressed genes may represent key molecular drivers in GBM malignant progression and provide promising candidates for further functional investigation.

### 3.2. Prediction of DEHP and Disease‐Target Genes

The toxicity of DEHP has been previously validated. Disease‐target genes were obtained from GeneCards and OMIM, while DEHP‐related genes were retrieved from CTD, SwissTargetPrediction, and the ChEMBL database. After duplicates were removed, the lists were intersected with the upDEGs and the UKB‐PPP dataset. Using Venn diagram mapping and analysis, 76 cross‐targets common to DEHP and GBM were identified (Figure 3A).

Figure 3(A) The intersection of genes associated with glioblastoma, DEHP‐related genes, UKB‐PPP, and upregulated genes from the single‐cell dataset, comprising a total of 76 genes. (B) Using the cytoHubba plugin in Cytoscape, the top 35 potential hub genes were identified using four algorithms: MCC, MNC, DMNC, and Degree. The intersection of the top‐ranked genes from these four algorithms was used for subsequent analysis. (C) Further analysis of the hub genes identified by the four algorithms above focused on their intersection, ultimately determining 24 hub genes.

**Figure:**
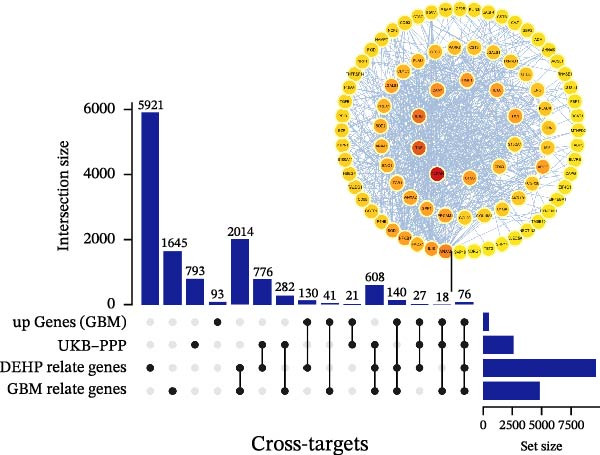
(A)

**Figure:**
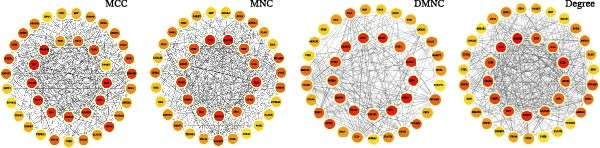
(B)

**Figure:**
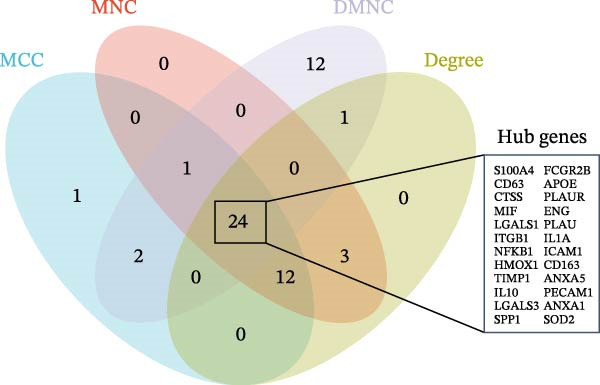
(C)

### 3.3. Construction and Analysis of the DEHP‐GBM Related PPI Network

As described in the Methods, we constructed a PPI network based on the 76 genes lying at the intersection of DEHP‐associated targets, GBM disease genes, macrophage upDEGs, and UKB‐PPP plasma proteins. The resulting STRING network (confidence score ≥0.4) comprised 89 nodes and 684 edges, indicating a highly interconnected set of proteins. Node size and color reflected Degree centrality, highlighting several densely connected proteins within the network. Using the CytoHubba plugin in Cytoscape, we then ranked nodes according to four centrality algorithms (Degree, DMNC, MNC, and MCC). For each algorithm, the top 35 genes were extracted, and the intersection of these four top‐ranked gene sets yielded 24 robust hub genes that were carried forward for subsequent MR and multi‐omics validation analyses (Figure 3B, C).

### 3.4. Analysis of the Causal Relationship Between Hub Genes and GBM Using MR

**Figure 4:**
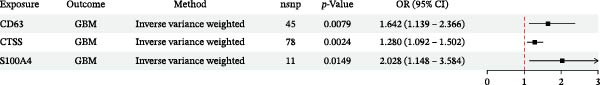
Mendelian randomization analysis of the causal relationship between hub genes and glioblastoma.

Upon in‐depth investigation of the 24 hub genes identified through network analysis, we found that only three met the threshold for statistical significance in their association analyses. Further MR analysis, using the IVW method, suggested that elevated expression of the *CD63*, *CTSS*, and *S100A4* genes was potentially causally associated with a significantly increased risk of GBM (Figure 4). Specifically, the estimated effect sizes indicated that each one‐unit increase in *CD63* expression corresponded to approximately a 64% increase in GBM risk (OR) = 1.64, 95% CI: 1.139–2.366); An elevation of one unit in *CTSS* expression correlated with a 28% heightened risk (OR = 1.28, 95% CI: 1.092–1.502). Similarly, a one‐unit increase in *S100A4* expression was associated with a 103% increase in risk (OR = 2.03, 95% CI: 1.148–3.584). Among these three genes, the association between CTSS and GBM risk exhibited the highest statistical significance value (smallest *p*‐value), whereas S100A4 demonstrated the largest effect size (Supporting Information 2: Table S1).

### 3.5. Multi‐Cohort Validation of GBM Marker Genes

The MR analysis (Figure 5A) revealed a robust positive causal association between the expression of *S100A4*, *CD63*, and *CTSS* and the risk of developing GBM, indicating that all three genes may function as genetic risk determinants for GBM onset. To further substantiate the reliability of this three‐gene signature, we next assessed its expression landscape across multiple independent cohorts. Using the TCGA‐GBM dataset integrated with GTEx as a primary validation cohort (Figure 5B), we observed a marked upregulation of *S100A4*, *CD63*, and *CTSS* in GBM tissues compared with normal brain samples (*p* < 0.05). We subsequently reinforced these findings through a multi‐cohort external validation by aggregating 11 independent GEO GBM datasets (GSE104267, GSE109857, GSE116520, GSE12657, GSE16011, GSE22866, GSE35493, GSE50161, GSE51146, GSE68848, and GSE7696), using the GCAS R package for dataset integration and analysis (https://github.com/WangJin93/GCAS). (Figure 5C–E). Strikingly, *S100A4* consistently showed the most pronounced differential expression, outperforming *CD63* and *CTSS* across datasets, underscoring its potential as a key driver and biomarker of GBM.

Figure 5(A) Scatter plots generated from Mendelian randomization analysis show a positive correlation between the three proteins, *CD63*, *CTSS*, and *S100A4*, and GBM risk, with the association for *S100A4* being the most significant. (B) Analysis of TCGA and GTEx datasets indicates that the expression levels of these three proteins were significantly higher in the GBM patient group than in the normal control group. (C–E) Comparative analysis across 11 GEO datasets revealed that, in the majority of datasets, *CD63* (excluding GSE104267 and GSE51146), *CTSS* (excluding GSE12657 and GSE7696), and *S100A4* (excluding GSE51146) were significantly overexpressed in the GBM group relative to the normal group. Statistical significance was defined as follows: ns, not significant; 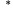 *p* < 0.05; 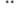 *p* < 0.01; 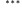 *p* < 0.001.

**Figure:**
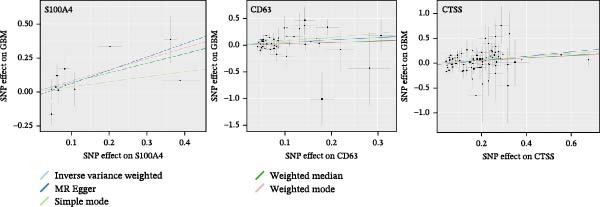
(A)

**Figure:**
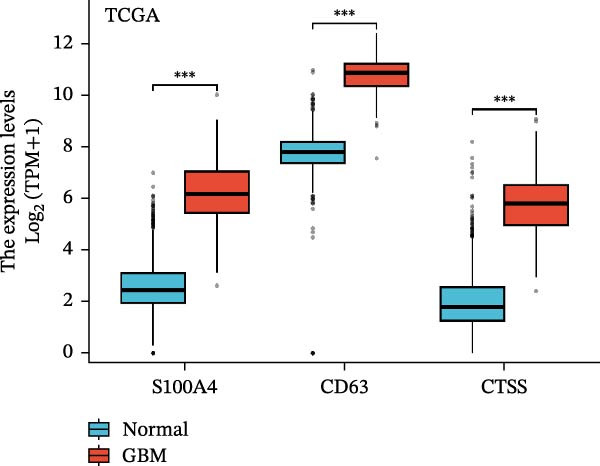
(B)

**Figure:**
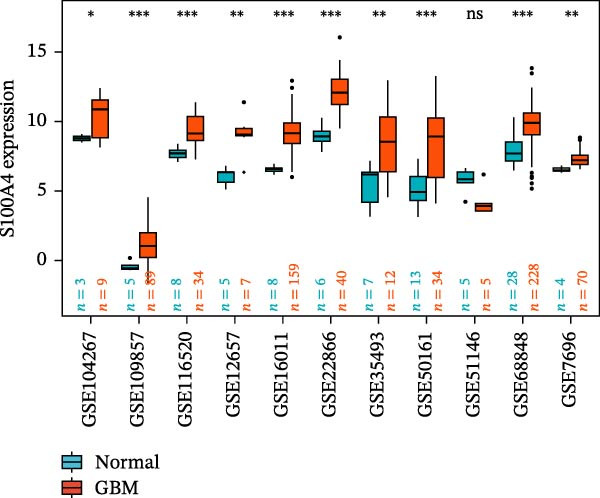
(C)

**Figure:**
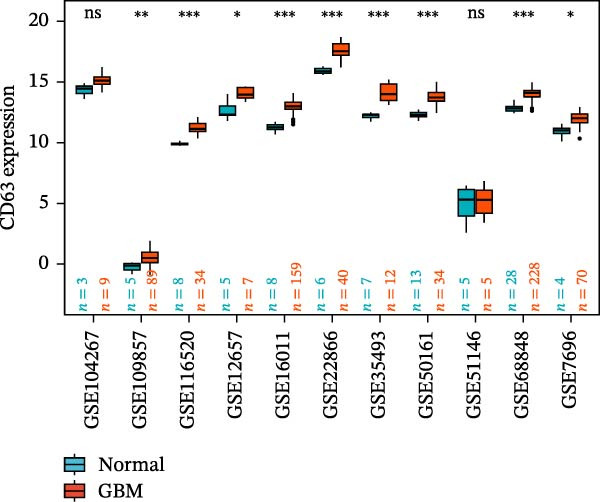
(D)

**Figure:**
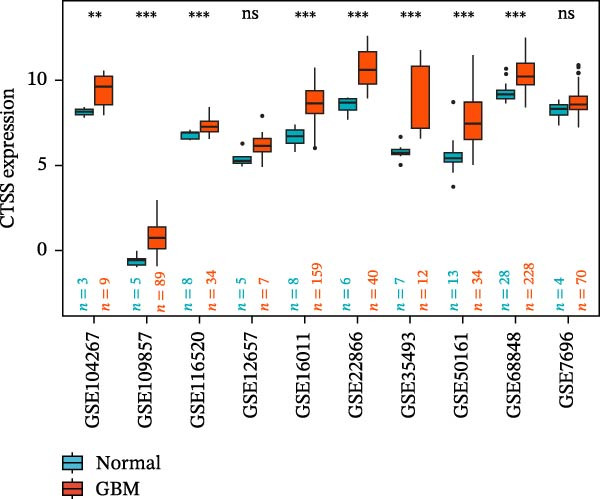
(E)

### 3.6. Analysis of Gene Expression Characteristics and Functional Regulation of Macrophages in the Immune Microenvironment of GBM Based on Single‐Cell Transcriptomic Data

Our single‐cell transcriptomic profiling of the GBM immune microenvironment showed that tumor‐associated macrophages (TAMs) were broadly transcriptionally activated, with coordinated upregulation of our core targets *CD63*, *CTSS*, and *S100A4*, as well as most other genes (Figure 2E). Further analysis demonstrated increased expression of these molecules across multiple cellular subpopulations (Figure 6A). Density plots confirmed that their expression was particularly enriched in GBM macrophages (Figure 6B), indicating spatial heterogeneity within the tumor.

Figure 6The gene expression patterns and functional characteristics of macrophages in the immune microenvironment of glioblastoma (GBM) based on single‐cell transcriptomic analysis. (A) Significant differences in expression of *S100A4*, *CD63*, and *CTSS* between GBM and control cell subpopulations. (B) The higher expression density of these genes in GBM macrophages. (C, D) The pseudotime trajectory of macrophages, indicating a transition from low to high invasiveness during tumor progression. (D) The dynamic changes in the expression of *S100A4*, *CD63*, and *CTSS* along the pseudotime axis. (E) The GO enrichment analysis, identifying key biological processes and molecular functions, including energy metabolism and cell adhesion, that are involved in macrophage function within the GBM immune microenvironment. Significance is defined as  ^∗^ *p* < 0.05,  ^∗∗^ *p* < 0.01,  ^∗∗∗^ *p* < 0.001,  ^∗∗∗∗^ *p* < 0.0001.

**Figure:**
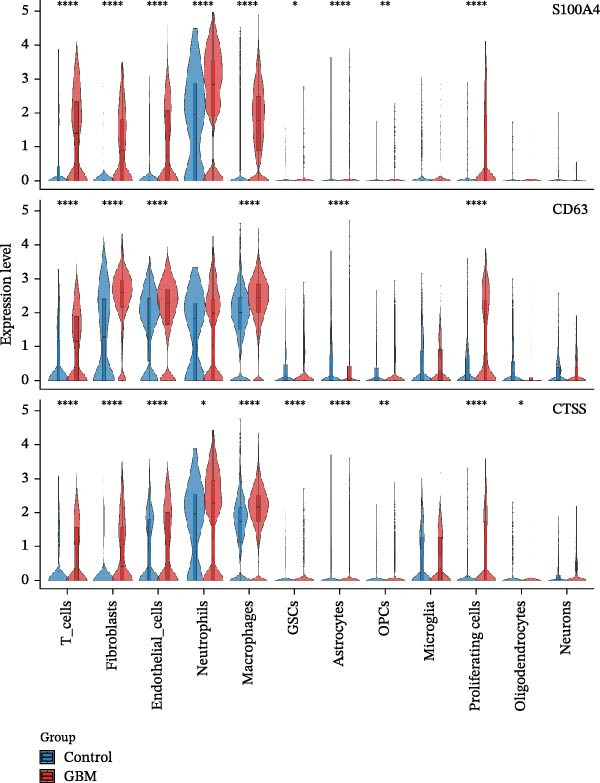
(A)

**Figure:**
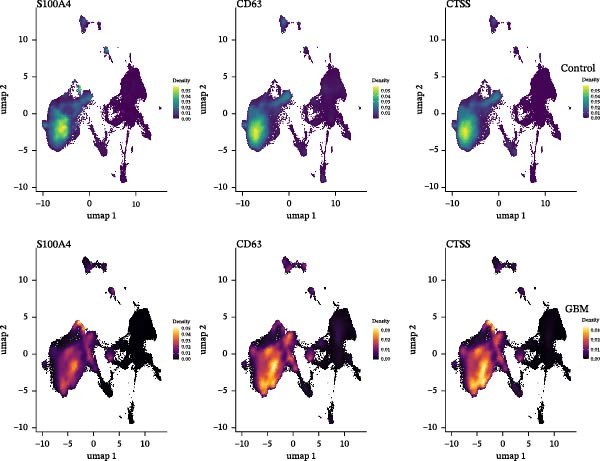
(B)

**Figure:**
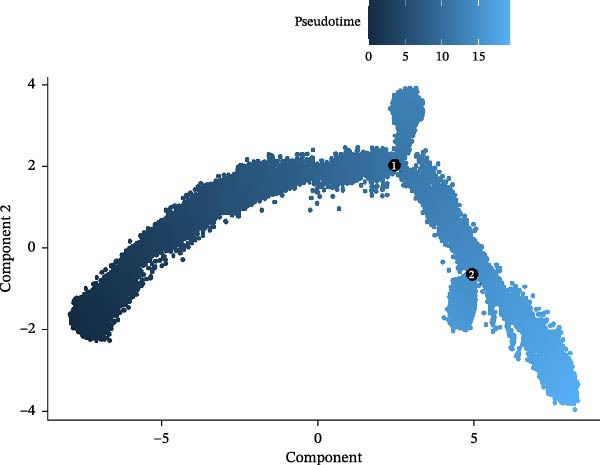
(C)

**Figure:**
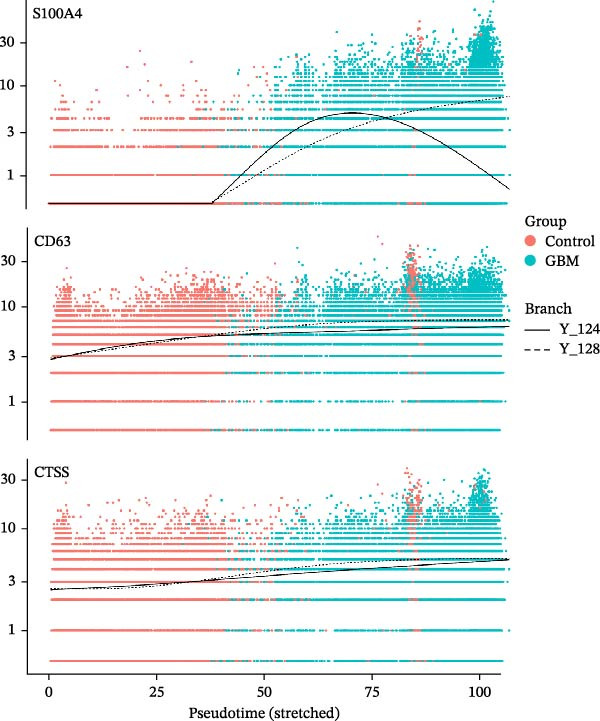
(D)

**Figure:**
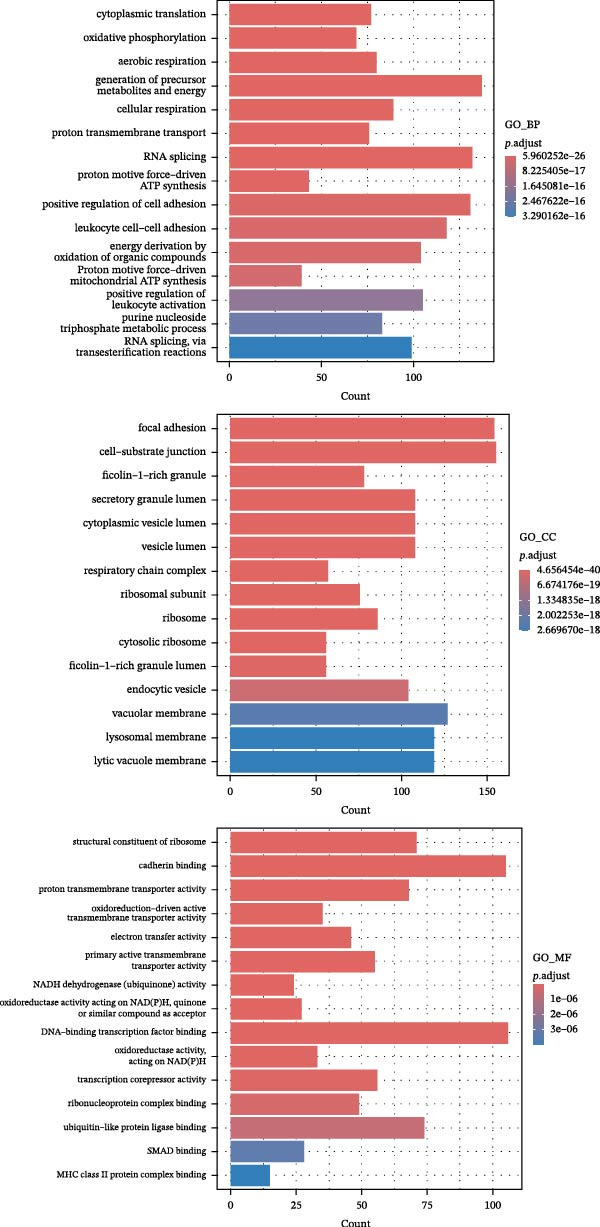
(E)

Pseudotime trajectory reconstruction mapped macrophage progression in GBM (Figure 6C). The trajectory suggested a shift from a low‐invasive to a high‐invasive state during tumor development, accompanied by substantial transcriptional changes, highlighting the involvement of macrophages in immune evasion and tumor progression. Monitoring the expression of *S100A4*, *CD63*, and *CTSS* along the pseudotime axis revealed dynamic expression patterns (Figure 6D). All three genes increased over the course of GBM progression. *S100A4* and *CD63* showed more pronounced elevations, suggesting roles in promoting invasiveness and immune escape, whereas *CTSS* displayed a distinct trend, suggesting a different mechanism of action.

GO enrichment analysis of macrophage differentially expressed genes revealed diverse functional alterations in the GBM microenvironment (Figure 6E). Enrichment of pathways related to energy metabolism, including cytoplasmic translation, oxidative phosphorylation, and aerobic respiration, indicated metabolic adaptation in TAMs. Enrichment in RNA splicing and transmembrane transport pathways suggested additional roles in regulating cellular signaling.

Cellular component analysis highlighted enrichment of focal adhesion and cell–matrix interaction complexes, consistent with macrophage involvement in tumor invasion. Enriched lysosomal and endocytic vesicle components suggested functions related to phagocytosis or immune modulation. Molecular function analysis identified enrichment for cadherin‐binding and DNA‐binding transcription factor activities, supporting roles in intercellular communication and transcriptional regulation. Increased representation of ribosomal components and ubiquitin ligase complexes reflected active protein turnover, while enrichment of proton transport, electron transfer, and NADH dehydrogenase activities further supported metabolic reprogramming. Additional enrichment of SMAD binding and related signaling pathways indicated that macrophages may influence cell proliferation and differentiation in GBM. Together, these data outline transcriptional and functional remodeling of macrophages in GBM, supporting their contribution to shaping the immune microenvironment and promoting GBM progression.

### 3.7. Molecular Docking

**Figure 7:**
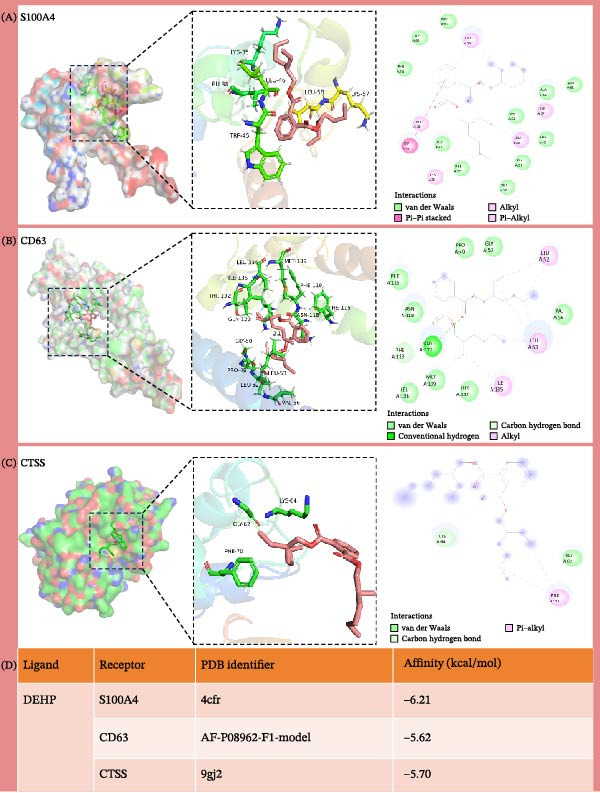
(A–C) The interactions between DEHP and the proteins CD63, CTSS, and S100A4, respectively. (D) A summary of these interactions, depicting the binding affinity of DEHP with each receptor protein, ranked as follows: S100A4 >CTSS >CD63, with S100A4 exhibiting the strongest affinity.

Molecular docking was employed in this study to evaluate the binding affinity between DEHP and three disease‐related target proteins, thereby substantiating DEHP’s potential toxicological effects (Figure 7A–C). The crystal structures of the aforementioned targets were obtained from the Protein Data Bank (PDB). The strength of these interactions was quantified by calculating binding free energy (Δ*G*), as more negative Δ*G* values indicate stronger binding affinity. Furthermore, the results indicated that DEHP formed stable complexes with all three target proteins (Figure 7D).

### 3.8. MD

To further assess DEHP‐target interactions, we performed a detailed analysis of binding free energies and structural dynamics from 100‐nanosecond MD simulations. Our analysis consistently identified S100A4 as the primary binding partner for DEHP. This conclusion is firmly supported by the calculated binding free energies, which were most favorable for the DEHP–S100A4 complex compared with the CD63 and CTSS complexes (Table 1, Figure 8A).

Figure 8MD simulation analysis of DEHP binding to S100A4, CD63, and CTSS. (A) Binding energy: S100A4 has the strongest affinity (Δ*G* = −37.22 kcal/mol), followed by CD63 and CTSS. (B) RMSD: CTSS/DEHP is the most stable; S100A4/DEHP shows increasing RMSD. (C) Ligand RMSD: CD63/DEHP stabilizes at ~2 Å, S100A4/DEHP and CTSS/DEHP at ~3 Å. (D) RoG: S100A4/DEHP is the most compact; CD63/DEHP shows greater fluctuation. (E) Hydrogen bonds: 2–4 bonds form between DEHP and each protein. (F) Conformational stability: CD63 and CTSS are flexible around residue 200; S100A4 is flexible at the N‐terminus.

**Figure:**
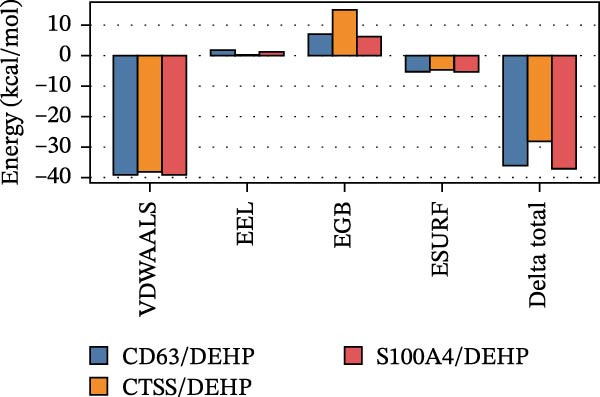
(A)

**Figure:**
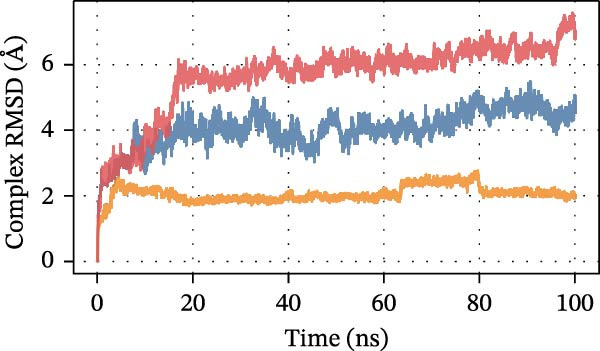
(B)

**Figure:**
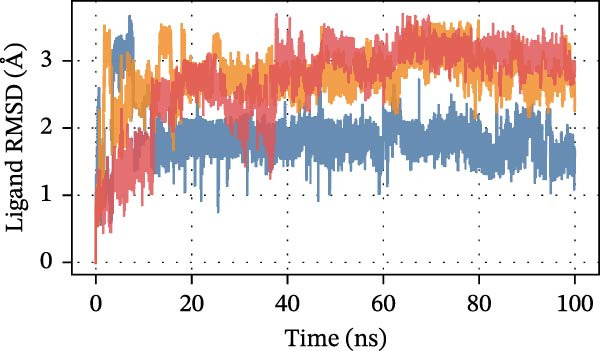
(C)

**Figure:**
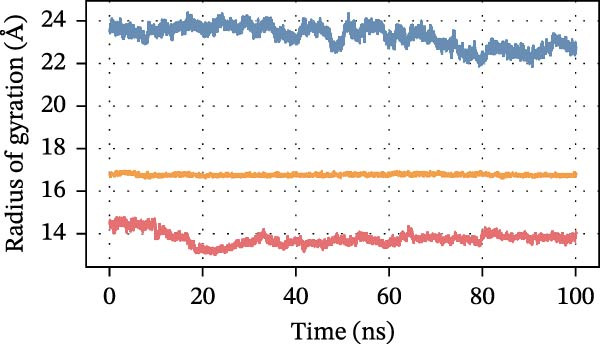
(D)

**Figure:**
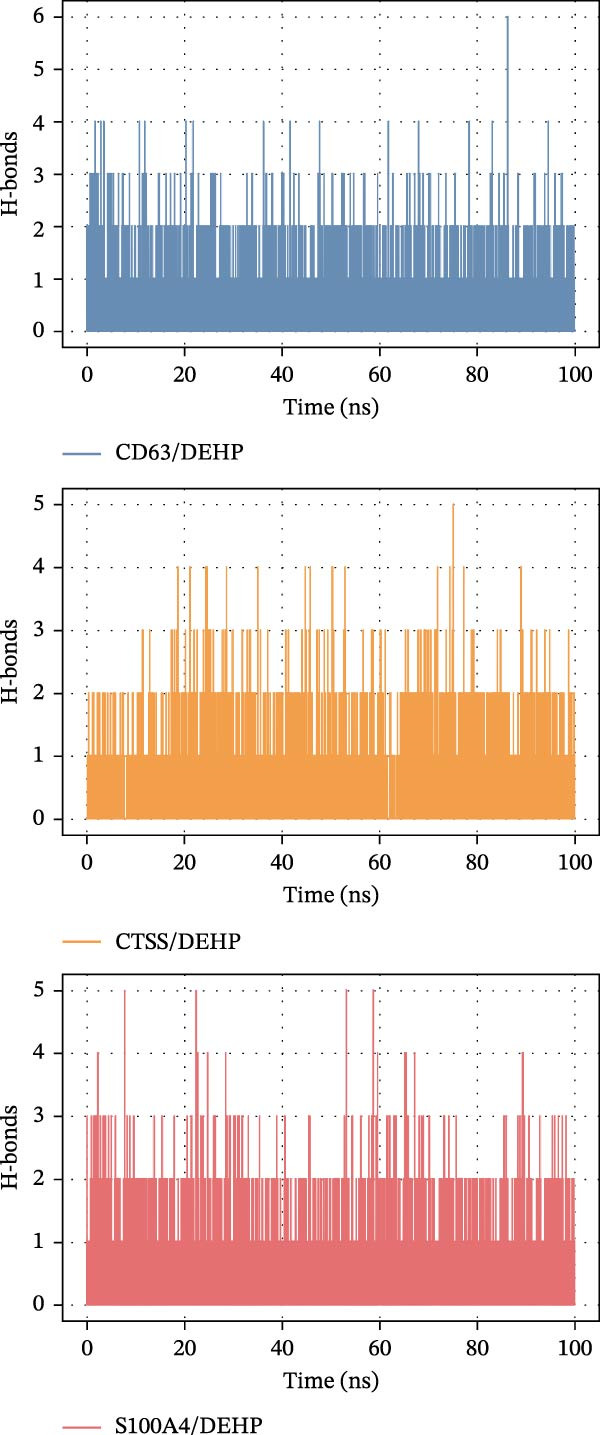
(E)

**Figure:**
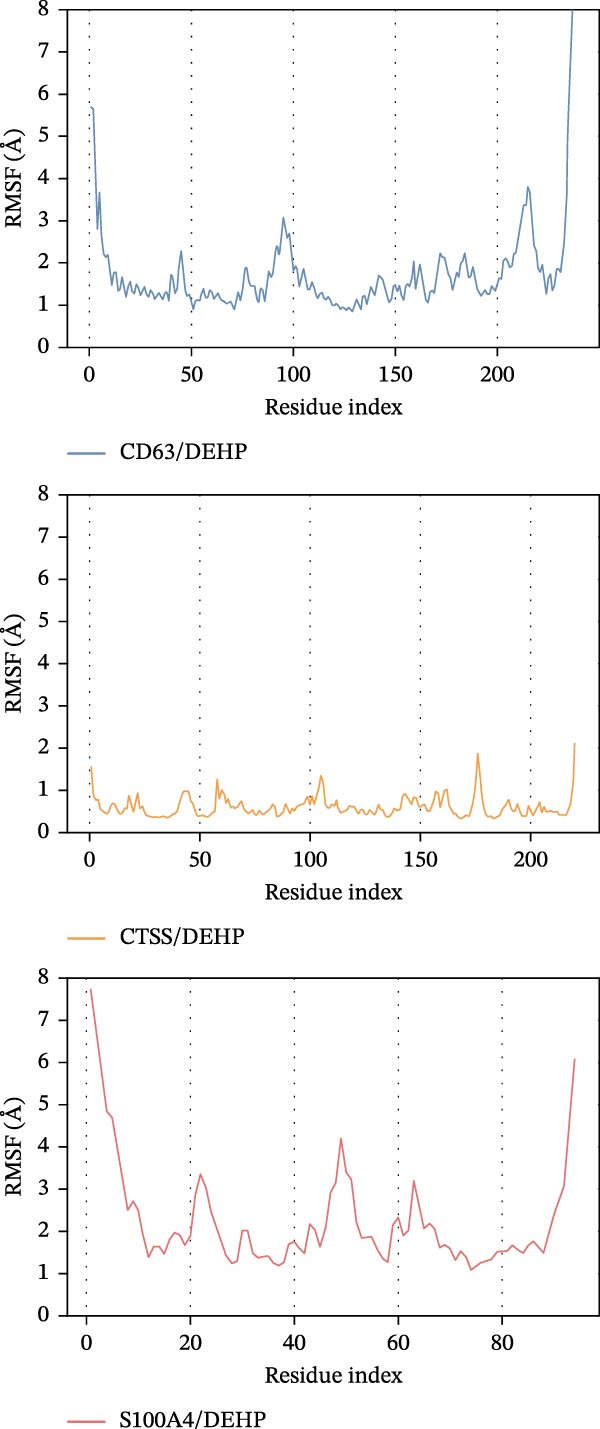
(F)

**Table 1:** The energy component analysis results characterizing the interactions between DEHP and the three proteins (CD63, CTSS, and S100A4).

Energy component	VDWAALS	EEL	EGB	ESURF	Delta total
CD63/DEHP	−39.05 ± 3.40	1.32 ± 1.65	6.98 ± 2.03	−5.31 ± 0.26	−36.06 ± 3.45
CTSS/DEHP	−38.13 ± 1.37	−0.22 ± 1.34	14.88 ± 1.58	−4.71 ± 0.23	−28.17 ± 1.30
S100A4/DEHP	−39.07 ± 2.39	1.15 ± 1.99	6.07 ± 2.01	−5.37 ± 0.38	−37.22 ± 2.61

*Note:* Binding energies for the interactions between DEHP and the three proteins (CD63, CTSS, and S100A4) are shown. All interactions are energetically favorable, with negative total energies indicating stable binding. The DEHP–S100A4 complex has the lowest energy, followed by CD63 and CTSS. The van der Waals forces are the main stabilizing factor, with contributions from electrostatic and other interactions. VDWAALS represents the van der Waals energy, EEL is the electrostatic energy, EGB is the solvation energy, and ESURF is the surface energy. DELTA total is the sum of these energies, indicating the overall binding strength.

Structural dynamics analysis provided further insights into the distinct interaction modes. The root‐mean‐square deviation (RMSD) analysis revealed that while the CTSS/DEHP complex maintained the most stable protein backbone (Figure 8B), the DEHP ligand itself achieved a stable binding pose within both the S100A4 and CTSS binding pockets after ~20 ns, as evidenced by ligand RMSD stabilization at around 3 Å (Figure 8C). Notably, radius of gyration (RoG) analysis demonstrated that the DEHP‐S100A4 complex maintained the most compact overall structure throughout the simulation (Figure 8D), suggesting a tight binding interface. This was complemented by the formation of 2–4 hydrogen bonds that sustained dynamic interactions between DEHP and each protein (Figure 8E).

Furthermore, root‐mean‐square fluctuation (RMSF) analysis revealed that S100A4 exhibited significant flexibility in its N‐terminal region (residues 40–80), with a prominent peak near residue 80 (Figure 8F). This inherent flexibility, combined with the observed conformational adjustments, suggests that DEHP binding may involve an induced‐fit mechanism with S100A4, in which structural adaptability facilitates the formation of a thermodynamically favored complex.

### 3.9. Mediation Analysis of S100A4 via Plasma Metabolites in GBM Association

**Figure 9:**
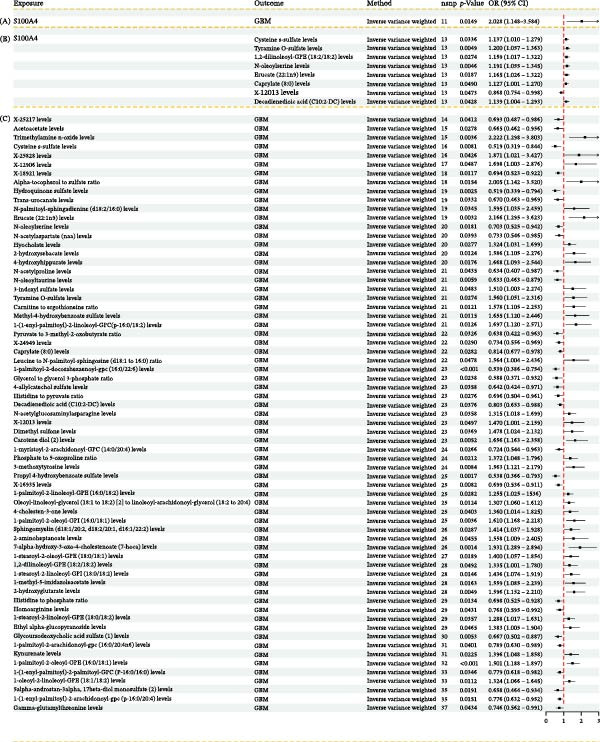
(A) Forest plot illustrating the association between S100A4 gene expression and the risk of GBM. (B) Forest plots showing the association between S100A4 gene expression and plasma metabolite levels. (C) Forest plot illustrating the associations between plasma metabolites and the risk of GBM.

Having identified the most stable S100A4 protein from MD simulations, we proceeded with a mediation analysis. First, we systematically employed the IVW method for MR analysis to assess the potential causal relationships between 1400 plasma metabolites and GBM. This examination identified 67 plasma metabolites associated with GBM risk (Figure 9C), of which 29 showed negative and 38 positive correlations. All analyses used 14–37 independent genetic instrumental variables (SNPs), and the CIs for all associations did not cross one, indicating statistical significance (*p* < 0.05). Specifically, elevated levels of the neuroprotective lipid 1‐palmitoyl‐2‐docosahexaenoyl‐GPC(16:0/22:6) were associated with a reduced risk of GBM (OR = 0.539, 95% CI 0.386–0.754); on the other hand, elevated levels of the pro‐inflammatory metabolite trimethylamine N‐oxide (TMAO) (OR = 2.222, 95% CI 1.298–3.803), the pro‐tumorigenic phospholipid 1‐palmitoyl‐2‐oleoyl‐GPE (16:0/18:1) (OR = 1.501, 95% CI 1.188–1.897), and the bile acid derivative 7‐alpha‐hydroxy‐3‐oxo‐4‐cholestenoate (7‐hoca) (OR = 1.931, 95% CI 1.289–2.894) significantly increased GBM risk. Furthermore, significant alterations were observed in key neurometabolites: a decrease in N‐acetylaspartate (NAA), a marker of neuronal integrity whose reduction reflects neuronal damage (OR = 0.733, 95% CI 0.546–0.985), an increase in the pro‐inflammatory neurotransmitter metabolite tyramine O‐sulfate (OR = 1.560, 95% CI 1.051–2.316), and accumulation of the immunosuppressive factor kynurenate (OR = 1.396, 95% CI 1.048–1.858). Collectively, these findings suggest that the development of GBM involves three key mechanisms: depletion of neuroprotective lipids, activation of pro‐tumorigenic phospholipid signaling, and establishment of a neuroinflammatory microenvironment (Supporting Information 2: Table S2).

Second, MR analysis confirmed that elevated S100A4 gene expression significantly increased the risk of GBM (OR = 2.028, 95% CI: 1.148–3.584) (Figure 9A) and causally altered the levels of nine plasma metabolites. Among these, the most pronounced increases were observed for tyramine O‐sulfate (OR = 1.200, 95% CI: 1.057–1.363) and N‐oleoylserine (OR = 1.191, 95% CI: 1.055–1.345), suggesting that S100A4 promotes tumorigenesis by modulating neurotransmitter metabolism and the endocannabinoid pathway. Concurrently, multiple lipid metabolites exhibited widespread upregulation, including erucate (22:1n9) (OR = 1.165, 95% CI: 1.026–1.322), and 1,2‐dilinoleoyl‐GPE (18:2/18:2) (OR = 1.159, 95% CI: 1.017–1.322), whereas the level of X‐12013 was significantly decreased (OR = 0.868, 95% CI: 0.754–0.998) (Figure 9B). These findings strongly suggest that S100A4 may drive GBM development by mediating the reprogramming of neuro‐lipid metabolism (Supporting Information 2: Table S3).

Based on the results from the two‐step analyses, we calculated the total effect (*β*_all_), the mediation effect (*β*_1_^∗^ *β*_2_), and the direct effect (*β*_direct_). According to the predefined criteria, a mediation effect accounting for more than 10% of the total effect was considered significant. In this study, we further investigated the impact of S100A4 on GBM, specifically evaluating the potential mediating roles of erucate (22:1n9) and tyramine O‐sulfate. The results demonstrated that the mediation effect of S100A4 on GBM via the erucate (22:1n9) pathway (17%) was significantly greater than that via the tyramine O‐sulfate pathway (11%), based on the proportion of the total effect. This finding suggests that among the potential mechanisms underlying S100A4’s influence on GBM, erucate (22:1n9) may play a more critical mediating role than tyramine O‐sulfate. This is further supported by its potentially higher signal transduction efficiency between the exposure factor (S100A4) and the outcome (GBM) (Table 2).

**Table 2:** Mediation analysis of S100A4 on GBM risk via plasma metabolites.

Exposure	Mediators	Outcome	*β*_all_	*β*_1_	*β*_2_	*β*_1_^∗^ *β*_2_	*β*_dir_	Mediation proportion (%)
S100A4	Erucate (22:1n9) levels	GBM	0.707	0.152	0.773	0.118	0.589	17
S100A4	Tyramine O‐sulfate levels	GBM	0.707	0.182	0.445	0.081	0.626	11

## 4. Discussion

This study systematically explores the potential association between environmental exposure to DEHP and the risk of GBM, as well as its putative molecular mechanisms. By integrating network toxicology, molecular docking, MR, MD simulations, and single‐cell transcriptomic analysis, we investigated how DEHP exposure may influence GBM risk. Specifically, we analyzed the regulation of key gene networks to gain insight into molecular pathways potentially involved in this process.

DEHP, a widely used plasticizer, has attracted significant attention due to its widespread environmental contamination, with annual production exceeding 8 million tons [45]. It is primarily released from plastic products, contaminating food, water, air, and household dust [46]. Human exposure occurs mainly through ingestion of contaminated food, inhalation, and dermal contact [47]. The molecular structure of DEHP confers endocrine‐disrupting properties, enabling it to accumulate in organisms and interfere with various biological processes readily. Although its specific gene targets are not yet fully elucidated, studies have demonstrated that DEHP significantly influences the expression of multiple genes, particularly those involved in metabolic processes and cellular stress responses, such as the cytochrome P450 genes (CYP35 family), among others [48]. This evidence provides valuable clues for understanding the potential biological effects of DEHP. In everyday life, DEHP exposure arises from contact with flexible PVC‐containing products such as food packaging, flooring materials, toys, and electrical cables, as well as from indoor dust and air, where it can reach relatively high concentrations. In medical settings, DEHP can leach directly from infusion sets, blood bags, dialysis tubing, and other PVC‐based devices into circulating blood, resulting in episodic high‐dose exposure in vulnerable patients such as neonates and critically ill adults. Typical internal exposure levels reported in biomonitoring studies fall within the low‐to‐moderate range for the general population but can be substantially higher in these high‐risk subgroups.

Previous in vivo and in vitro studies have linked DEHP exposure to various diseases, with particular concern regarding its carcinogenic potential. Research has indicated that DEHP may be associated with the initiation and progression of multiple cancers, including colorectal, liver, breast, prostate, pancreatic, and bladder cancer [49–52]. These findings provide a preliminary scientific foundation for further investigating the role of DEHP in GBM. Furthermore, the mechanisms by which DEHP influences cancer development may involve multiple pathways, including the activation of nuclear receptors (PPARα, ERα, and AhR), disruption of redox homeostasis, induction of epigenetic modifications, and promotion of acquired drug resistance in tumor cells [53]. These mechanisms may contribute to the development and progression of various tumors, including GBM. Therefore, a further in‐depth investigation into the potential relationship between DEHP and GBM, particularly its possible impact on relevant biomarkers, is essential to better understand GBM pathogenesis and to explore potential diagnostic and therapeutic strategies.

Our findings highlight CD63, CTSS, and S100A4 as critical drivers of GBM pathogenesis and powerful predictors of clinical outcomes. CTSS, a cysteine protease, is strongly linked to the malignant progression and poor prognosis of GBM [54]. It achieves this by potentially enhancing tumor cell invasion and migration, promoting proliferation, and modulating the extracellular matrix and signaling pathways [55]. CD63, a cysteine‐rich transmembrane protein, participates in fundamental cellular processes such as proliferation, migration, adhesion, differentiation, and motility [56, 57]. Notably, CD63 expression levels positively correlate with GBM malignancy grade, suggesting its potential to promote tumor growth and metastasis by modulating the tumor microenvironment, regulating cell adhesion, and modulating signal transduction. These findings position CD63 as a potential therapeutic target. Furthermore, S100A4 exerts its function by activating signaling pathways such as the Wnt/β‐catenin and NF‐κB pathways [58, 59]. A study on GBM patients revealed that high S100A4 expression exhibits oncogenic activity and can serve as a target for immunotherapy [60], while also promoting tumor migration and angiogenesis [61]. Therefore, S100A4 is considered a valuable prognostic biomarker due to its role in tumor progression. In summary, CD63, CTSS, and S100A4 are not only key nodes for understanding the complex pathological mechanisms of GBM but also offer promising directions for developing targeted therapeutic strategies aimed at these molecules. These strategies have the potential to improve patient outcomes.

Beyond macrophages, S100A4 expression is not restricted to the myeloid compartment. Previous single‐cell and histopathological studies in glioma have reported S100A4‐positive malignant cells as well as perivascular stromal or fibroblast‐like populations, where S100A4 can regulate vascular remodeling and invasive behavior [60–62]. These observations suggest that S100A4 may coordinate a multicellular network involving TAMs, neoplastic cells, and stromal elements rather than acting within a single cell type. Our macrophage‐centered scRNA‐seq analysis should therefore be viewed as highlighting one critical node within a broader S100A4‐driven communication axis. Future work integrating multi‐compartment single‐cell transcriptomics and spatial profiling will be required to dissect the directionality and functional consequences of this crosstalk.

To investigate the interaction between DEHP and these key proteins, we performed molecular docking to evaluate DEHP’s binding affinities for CD63, CTSS, and S100A4. The results indicated that DEHP can form stable complexes with these proteins, with S100A4 exhibiting the strongest binding affinity, followed by CTSS and CD63. The calculated binding free energy for the DEHP‐S100A4 complex (Δ*G* = −37.22 kcal/mol) suggests a high affinity interaction in silico. While this value indicates strong binding under computational conditions, its relevance to physiological scenarios requires careful consideration. The nanomolar to low micromolar concentrations of DEHP found in human plasma suggest that moderate‐to‐high affinity binding (in the µM–nM range) could be biologically relevant. Our computed Δ*G*, if translated to a dissociation constant (Kd), would imply a very strong (likely sub‐nanomolar) binding, which may exceed what is expected for a diffuse environmental interaction. This discrepancy highlights a limitation of the computational model and suggests that the actual interaction in a complex biological milieu might be weaker or require cofactors. Future studies should aim to experimentally determine the binding affinity and assess whether it falls within a range plausible for the observed plasma DEHP concentrations. Further MD simulations (conducted over a 100‐ns timescale) assessed the stability of the DEHP‐protein complexes. The simulations revealed that S100A4 and CTSS maintained structural stability, whereas CD63 underwent conformational changes, which indicates differences in the stability of their interactions with DEHP. The calculated binding energies further supported the docking results, and S100A4 displayed the lowest binding energy, further reinforcing its role as a key target in the context of DEHP exposure. Interestingly, the RMSF analysis indicated significant flexibility in the N‐terminal region of S100A4. S100A4 is known to exist in multiple conformational states, including dimeric and tetrameric forms, which can influence its interaction with partners like RAGE and non‐muscle myosin IIA, thereby modulating its pro‐metastatic functions. Our MD simulations suggest that DEHP binding may stabilize a particular conformation of S100A4, potentially one that favors interactions promoting tumor cell motility and invasion. This stabilization of a “pro‐metastatic conformation” by an environmental toxicant represents a novel hypothetical mechanism that warrants experimental investigation. These results align with the existing literature, which emphasizes the importance of protein stability in drug development and toxicological assessments. Moreover, they highlight the potential relevance of DEHP interactions with these proteins in GBM development. Validation using multi‐cohort data has further supported these observations.

Our mediation analysis identified erucic acid (erucate, 22:1n9) as a significant mediator, accounting for ~17% of the effect of S100A4 on GBM risk. The biological plausibility of erucic acid as a specific mediator in this context is supported by several lines of evidence. First, erucic acid is a very‐long‐chain monounsaturated fatty acid whose metabolism and accumulation have been linked to cellular stress and lipotoxicity. More directly, DEHP exposure has been shown to disrupt fatty acid metabolism and homeostasis. Studies indicate that DEHP and its metabolites can interfere with peroxisomal β‐oxidation, the primary pathway for degrading very‐long‐chain fatty acids like erucic acid. Inhibition of this pathway by DEHP could lead to the accumulation of erucic acid and other long‐chain fatty acids. Furthermore, DEHP activation of PPARα can alter the expression of genes involved in lipid synthesis and breakdown, potentially skewing the fatty acid profile. Therefore, it is plausible that DEHP exposure, through its documented effects on lipid metabolism, could contribute to elevated erucic acid levels, which our MR analysis then links to GBM risk via an S100A4‐associated pathway. This provides a coherent, albeit preliminary, toxicological link between the exposure (DEHP), the molecular hub (S100A4), and the metabolic phenotype (erucic acid accumulation).

This study has several limitations that should be acknowledged. First, and foremost, the research is primarily based on computational predictions, bioinformatics analyses, and genetic epidemiology. The proposed DEHP‐S100A4 interaction and its downstream effects lack direct experimental validation in cellular or animal models of GBM. Consequently, the causal chain and mechanistic details remain hypothetical. Future work must include in vitro assays to test if DEHP exposure alters S100A4 expression, stability, or function in GBM‐relevant cells (e.g., macrophages, glioma stem cells), and in vivo studies to confirm its impact on tumor growth and metabolism. Second, while MR provides evidence for a genetic causal link between S100A4 and GBM, it does not prove that environmental DEHP exposure causes GBM via upregulating S100A4. These are distinct levels of evidence that should not be conflated. Third, the datasets used, particularly for GWAS and metabolomics, are predominantly from European ancestry populations, limiting the generalizability of the findings to other ethnic groups. The applicability across different geographical regions and populations needs further investigation. Fourth, sample sizes for some analyses (e.g., the FinnGen GBM cohort) are moderate, and integrating multisource data may introduce unresolved batch effects. Finally, the clinical relevance of the identified biomarkers and the feasibility of targeting this axis for intervention require extensive future validation.

Despite these limitations, our findings have potential public health and clinical implications. DEHP remains a ubiquitous environmental contaminant, and our study adds to the concern about its potential role in carcinogenesis, specifically in the brain. If the DEHP‐S100A4‐lipid axis is validated, it would highlight an actionable environmental risk factor for a devastating cancer. This underscores the importance of public health policies aimed at reducing DEHP exposure, especially in vulnerable settings like healthcare and for children, and promotes the development of safer alternative plasticizers. Clinically, S100A4 and its associated lipid signature (e.g., erucic acid) could be explored as biomarkers for identifying individuals with environment‐associated GBM subtypes or for monitoring intervention efficacy. Furthermore, S100A4 itself emerges as a potential therapeutic target to mitigate the oncogenic effects of environmental exposures. However, these translational steps are contingent upon robust experimental confirmation of the mechanisms proposed here.

## 5. Conclusion

In summary, this integrative multi‐omics study supports a potential link between the environmental contaminant DEHP and GBM risk, proposing the “DEHP–S100A4‐lipid axis” as a potential pathogenic pathway. By synergizing network toxicology, MR, and single‐cell transcriptomics, we identified and validated S100A4 as a key mediator, demonstrating stable binding with DEHP in silico and a causal relationship with GBM risk at the genetic level. Further mediation analysis revealed that S100A4 promotes tumorigenesis, in part, by reprogramming lipid metabolism, with metabolites such as erucic acid accounting for up to 17% of the effect. Our findings extend the mechanistic understanding of GBM by delineating how a prevalent environmental exposure may converge on a defined molecular pathway to drive oncogenesis. At the same time, it should be noted that the evidence provided here is primarily computational and genetic‐epidemiologic and thus should be regarded as hypothesis‐generating rather than definitive proof of causality. This work not only provides a valuable scientific perspective but also underscores the importance of environmental risk factors in neuro‐oncology, highlighting potential targets for preventive strategies. Future in vitro and in vivo studies will be essential to verify whether DEHP directly modulates S100A4 expression and function in the brain, to clarify the downstream lipid metabolic changes in GBM models, and to determine whether intervening on this axis can meaningfully reduce DEHP‐associated tumor risk in translational and clinical contexts.

## Author Contributions

**Shasha Tan**: investigation, formal analysis, visualization, writing – original draft. **Zhou Li**: investigation, data curation, validation. **Zhenjiang Du**: data curation, software, methodology. **Jinliang You**: software, methodology. **Lichun Qiao**: methodology, formal analysis, data curation. **Long Zhao**: conceptualization, investigation. **Binbin Yang**: software, validation, methodology. **Xiaoping Tang**: methodology, writing – review and editing. **Sajjad Muhammad**: supervision, writing – review and editing. **Hongjun Liu**: conceptualization, supervision, project administration, writing – review and editing.

## Funding

No funding was received for this manuscript.

## Ethics Statement

The publicly accessible, anonymized databases did not require ethical approval or informed consent for this research, which complied with relevant ethical guidelines.

## Conflicts of Interest

The authors declare no conflicts of interest.

## Supporting Information

Additional supporting information can be found online in the Supporting Information section.

## Supporting information


**Supporting Information 1** STROBE‐MR checklist: Reporting guideline checklist for Mendelian randomization studies.

**Supporting Information 2** Table S1: Three key genes in glioblastoma multiforme: associations and sensitivity analysis. Table S2: Plasma metabolites in GBM: associations and sensitivity analysis. Table S3: S100A4 in plasma metabolites: associations and sensitivity analysis.

## Data Availability

All data are sourced from public databases. This study utilized data from multiple sources. Plasma proteomics data were sourced from the UK Biobank Pharma Proteomics Project (UKB‐PPP), which quantified circulating protein abundances in ~54,000 participants using the Olink Explore platform, enabling genome‐wide proteogenomic analyses across thousands of protein targets. The genetic and phenotypic GWAS data were obtained from the FinnGen database (release R12), which includes data from 406 GBM patients and 378,749 control samples. Furthermore, GWAS data on 1400 plasma metabolites, with identifiers ranging from GCST90199621 to GCST90201020, were sourced from a 2023 study published in the GWAS Catalog and limited to individuals of European ancestry.
